# Facilitating professional liaison in collaborative care for depression in UK primary care; a qualitative study utilising normalisation process theory

**DOI:** 10.1186/1471-2296-15-78

**Published:** 2014-05-01

**Authors:** Nia Coupe, Emma Anderson, Linda Gask, Paul Sykes, David A Richards, Carolyn Chew-Graham

**Affiliations:** 1 Centre for Primary Care, Institute of Population Health, Williamson Building, Oxford Road, University of Manchester, M13 9PL, Manchester, UK; 2 School of Experimental Psychology, University of Bristol, Bristol, BS8 1TU, UK; 3 University of Exeter Medical School, Haighton Building, St Luke’s Campus, Heavitree Road, EX1 2 LU, Exeter, UK; 4 Primary Care and Health Sciences, Keele University, Staffordshire, ST5 5BG, UK

## Abstract

**Background:**

Collaborative care (CC) is an organisational framework which facilitates the delivery of a mental health intervention to patients by case managers in collaboration with more senior health professionals (supervisors and GPs), and is effective for the management of depression in primary care. However, there remains limited evidence on how to successfully implement this collaborative approach in UK primary care. This study aimed to explore to what extent CC impacts on professional working relationships, and if CC for depression could be implemented as routine in the primary care setting.

**Methods:**

This qualitative study explored perspectives of the 6 case managers (CMs), 5 supervisors (trial research team members) and 15 general practitioners (GPs) from practices participating in a randomised controlled trial of CC for depression. Interviews were transcribed verbatim and data was analysed using a two-step approach using an initial thematic analysis, and a secondary analysis using the Normalisation Process Theory concepts of coherence, cognitive participation, collective action and reflexive monitoring with respect to the implementation of CC in primary care.

**Results:**

Supervisors and CMs demonstrated coherence in their understanding of CC, and consequently reported good levels of cognitive participation and collective action regarding delivering and supervising the intervention. GPs interviewed showed limited understanding of the CC framework, and reported limited collaboration with CMs: barriers to collaboration were identified. All participants identified the potential or experienced benefits of a collaborative approach to depression management and were able to discuss ways in which collaboration can be facilitated.

**Conclusion:**

Primary care professionals in this study valued the potential for collaboration, but GPs’ understanding of CC and organisational barriers hindered opportunities for communication. Further work is needed to address these organisational barriers in order to facilitate collaboration around individual patients with depression, including shared IT systems, facilitating opportunities for informal discussion and building in formal collaboration into the CC framework.

**Trial registration:**

ISRCTN32829227 30/9/2008.

## Background

Depression is an increasingly common mental health problem worldwide, set to become the second most debilitating condition in the world by 2020 [1]. It is characterised by a wide range of symptoms, most notably low mood, and has been found to be more detrimental to health than other physical long term conditions [2]. Depression goes undetected, and consequently untreated, in around half of the patients with depression attending primary care [3].

Many countries have initiated quality improvement programmes for depression including case-finding for depression in primary care [4], clinical guidelines [5,6] and organisational interventions to improve the management of depression [7]. However, a lack of access to resources has been identified as a source of frustration for GPs [8-10] and poor communication between generalist and specialist mental health practitioners in managing patients with depression, attributed to organisational barriers, has also been identified as problematic [11].

Collaborative care (CC) is an organisational framework derived from the chronic care model that aims to improve patient care by increasing professional communication and providing a more structured approach to depression management [12]. The essential characteristics of CC are given in Table 1.

**Table 1:** **The collaborative care framework (Gunn et al, 2006) **[12]

*Multi*-*professional approach to patient*	Care provided by a case manager working with the family doctor under weekly supervision from specialist mental health medical and psychological therapies clinicians.
*Structured management plan*	Medication support and brief psychological therapy
*Scheduled patient follow*-*ups*	Proactive care
*Enhanced inter*-*professional communication*	Patient-specific written feedback to family doctors via electronic records and personal contact

Collaborative care is effective for the management of depression [13], with the majority of evidence coming from the USA where it has been found to be effective in improving outcomes of depression [14,15] and depression in the presence of long term physical health conditions (LTCs) [16]. Recently, evidence has established that the positive effects of collaborative care generalise to countries such as Chile [17], India [18] and the UK [19], where we conducted a series of feasibility studies [20-23] culminating in the Collaborative Care for Depression Trial (CADET) [19], a two-arm cluster-randomised controlled trial (RCT) of CC compared to usual care for patients with depression in primary care, based within three UK sites (Bristol, Manchester and London). Recruitment of practices to the trial was facilitated by the Mental Health Research Networks, and then by members of the research team. Senior researchers in each site visited practices to introduce the study, the concept of collaborative care and the role of the practices in recruiting patients.

CADET demonstrated collaborative care improves depression immediately after treatment compared to usual care, with effects that persisted at 12 months follow up, and is preferred by patients over usual care [19].

The collaborative care intervention in CADET, based on the Gunn model, comprised case management by specifically trained primary care mental health workers supervised by mental health specialists. The case managers (CMs) delivered a complex intervention comprising symptom assessment and goal-setting, behavioural activation (BA), and medication management (MM). The CC framework encouraged liaison between the CMs and the patient’s GP. CMs had regular contact (between 6 and 12 contacts) with patients over a four month period, with an initial face to face assessment and the remainder by telephone.

Whilst CC has a solid evidence base, in order for it to be implemented into routine practice we need to understand the processes involved and work required for its development, implementation and sustainability, with the overall aim of understanding how the intervention can be ‘normalised’ into practice. In particular, although the clinical components seem robust, we need to know more about how best to facilitate collaboration between case managers, primary care professionals and mental health specialists. One theoretical model of implementation - Normalization Process Theory (NPT) (see Table 2) [24] – is concerned with understand the dynamics of implementing, embedding, and integrating a new technology or complex intervention- such as Collaborative Care within a healthcare system. NPT has four components which can be used to evaluate implementation of complex interventions. *Coherence* encompasses whether the intervention makes sense to, and is perceived to be of value to the relevant participants, and whether it fits with the goals and activities of the organisation. *Cognitive participation* considers whether participants will be prepared to invest in the new intervention. *Collective action* asks what effect the intervention will have on current work, and whether it is consistent with existing practices and Reflexive monitoring asks how participants perceive the intervention once it has been in place for a while.

**Table 2:** **Normalisation process theory has four key elements** (**from May and Finch**, **2009**, **pp**: **542**-**545 **[24] **and **http://www.normalizationprocess.org**)**

**Coherence:** a set of ideas about the meaning, uses and utility of a practice, (defined as an ensemble of beliefs, behaviours, and acts that manipulate or organize objects and others), which hold the practice together and make it possible to share and enact it.	
● *This is the****sense***-***making work****that people do individually and collectively when they are faced with the problem of operationalizing some set of practices*	
● **Cognitive participation:** the symbolic and real enrolments and engagements of human actors that position them for the interactional and material work of collective action.	
● *This is the ****relational work ****of that people do to build and sustain a community of practice around a new technology or complex intervention*	
**Collective action:** the chains of interactions which are the site of mental and material work to organise and enact practice which might include reshaping behaviours or actions, employing objects or artefacts, or reorganising relationships and contexts.	
● *This is the ****operational work ****that people do to enact a set of practices*, *whether these represent a new technology or complex healthcare intervention*.	
**Reflexive monitoring:** the continuous evaluation, both formally and informally, of implementation processes by participants, which may involve judgements about the utility and effectiveness of a new practice with reference to socially patterned and institutionally shared beliefs	
● *This is the ****appraisal work ****that people do to assess and understand the ways that a new set of practices affect them and others around them*	

### Objective

Building on a previous process evaluation which used the Normalisation Process Model (NPM) [25] to identify the work required to implement CC for depression [22], we aimed to identify barriers and facilitators to the successful implementation of CC into UK primary care.

## Methods

The CADET trial and nested qualitative study was given a favourable ethics opinion by the South West Research Ethics Committee on behalf of the NHS National Research Ethics. Site specific approvals were subsequently obtained for each NHS/Primary Care Trusts.

### Recruitment and sampling

We interviewed all six CMs and five supervisors involved in delivering and supervising CC in CADET across the three sites, along with a purposive sample of GPs whose practices were participating in the CADET trial [26]. Case managers were mental health workers working within either local primary care NHS organisations or third sector health and social care services. Supervisors were all clinical academics and co-investigators involved in the CADET trial.

We sampled GPs purposively based on location, GP surgery, years of experience and practice demographics. We ceased recruitment when category saturation of data was achieved. We used flexible topic guides for all interviews with open-ended questions to encourage discussion. Interviews with case managers and supervisors were conducted face-to-face by NC in their place of work from September 2010 to January 2011; and GPs over the telephone between March and May 2011 by NC, PS and EA. Telephone interviews were offered to GPs in order to cause minimum disruption to their working day. All interviews were audio-recorded with consent, anonymised and transcribed verbatim.

### Analysis

The transcripts from each interview formed the data. We used an iterative approach using constant comparison techniques [27] and topic guides which we reviewed and adapted after each interview following discussions between authors as the study progressed, allowing for emerging themes to be incorporated into the topic guides. Authors CCG, NC, EA and PS conducted an initial thematic analysis and coding [28] independently at first, and themes were agreed through discussion between researchers of different professional backgrounds (general practice, nursing, psychology). Following the thematic analysis we conducted a further theory-driven analysis of the data guided by the four main constructs of NPT (coherence, cognitive participation, collective action and reflexive monitoring). Authors CCG, NC, EA and LG conducted this analysis individually, and the final analysis was agreed through discussion, with data being tabulated to illustrate the four constructs of NPT. Disconfirmatory evidence was sought in the data throughout the analysis.

## Results

The demographics of CMs and supervisors are not been included to ensure anonymity of participants. GP demographics can be seen in Table 3.

**Table 3:** Demographics of GPs interviewed

**GP**	**Gender**	**Years of experience as GP**	**Practice population**	**Practice size**	**IMD rank**	**CADET recruitment figures**	**Actively involved in commissioning**
**GP001**	Female	25 years	Afro-Caribbean, Asian, Eastern European and Turkish, long stay, suburban.	14000	4339	16	No
**GP002**	Male	17 years	50% Caucasian 50% Asian, urban, deprived, socio-economic mix, many family residents.	2800	2938	12	Yes
**GP003**	Male	39 years	Urban, mixed social class - less deprived (group 1 & 2).	8000	26048	13	No
**GP004**	Male	31 years	Urban, mixed social class - less deprived (group 1 & 2).	8000	26048	13	No
**GP005**	Female	25-26 years	Almost totally white, not deprived, urban edges/semi-rural. Core of family-based patients.	2350	14588	11	No, but is mental health lead for PCT
**GP006**	Male	28 years	High deprivation, 5-10% Asian population, 1/3 transient, 2/3 settled (lots of families), over-represented mental health comp to other practices.	3500	1128	9	No
**GP007**	Female	21 years	High deprivation, white British, high unemployment, many patients with smoking-related illnesses.	6000	317	13	Yes in future
**GP008**	Male	15 years	Afro-Caribbean, Asian, Eastern European and Turkish, long stay, suburban.	14000	4339	16	No
**GP009**	Male	14 years	Younger population, high turnover, Eastern Europeans, Afro-Caribbean, South Asian, Minority Far East, higher than ‘normal’ mental health issues.	7500	1809	8	Yes but resigning due to political nature
**GP010**	Male	30 years	Diverse, multi-ethnic. Top 10% most deprived areas in country. A lot of mental health issues.	8000	3428	12	Not for last 18 months
**GP011**	Male	18 years	Two branches, slightly different demographics in each. One has new Eastern European immigrants; other has significant Asian and African Caribbean. Suburban teaching/training practice.	8300	9601/128182 branches	12	Not any more
**GP012**	Male	7 years	Same surgery as above. This GP says is inner city practice. Lots of people with English as second language. Mobile patient population (high turnover).	8300	9601/128182 branches	12	Not asked
**GP013**	Male	17 years	Mainly white males aged 25-35, a few Asian, Chinese and Black people.	7600	8179	16	No
**GP014**	Female	10 years	Mainly white males aged 25-35, a few Asian, Chinese and Black people.	7600	8179	16	No
**GP015**	Male	22 years	Majority white British, very few black and minority ethnic groups.	7750	317	13	No

The initial thematic analysis is summarised in Table 4, with some illustrative data given.

**Table 4:** Initial thematic analysis

**Main theme**	**Sub**-**themes**	**Illustrative data**
Recognizing the need for change	GPs’ understanding of current services	“*Theoretically we have access to counselling services. There is a group commissioned by the PCT called* [*names team*] *which I think has changed over the years from being a purely sort of counselling service to one with a range of psychological services*” GP011
	Limited access to services	“…*psychological services as opposed to psychiatric acute services are dire locally*, *absolutely dire*…*we have such limited access*, *there*’*s just such a burden of* …*mild to moderate psychiatric illness and that isn*’*t well catered for at all*” GP001
	Reflections on the past	“*The structure*, *I think*, *the way we used to work in the old days we used to work collaboratively anyway*, *which was really good*, *erm*, *but we haven*’*t got that structure now*, *so it*’*s about number crunching really*, *you know*, *in terms of referrals coming through to you*, *and being based at*… *a main health centre where they have to come to you*” CM102
Operationalising collaborative care	Understanding collaborative care	“*I was re*-*reading the protocol for this session* (*interview*) *and thinking*, *should I have been doing more with GPs*? *Talking with them more about medication*? *So I thought*, *maybe I*’*ve done something kind of wrong and not quite completely as collaborative as I could have been*, *I think I probably could*’*ve done more*” CM106
	Delivering the intervention	“*I didn*’*t really understand collaborative care*; *I*’*ll be quite honest*… *I didn*’*t know what collaborative care was*, *although I could have had a guess. Collaborative care would have meant care that involved both myself and someone else*, *if you see what I mean*” GP004
		“*It*’*s a better experience for the therapist*, *I*’*ve kind of had a really positive experience of CADET*, *which I think if I*’*d purely had experience of IAPT I wouldn*’*t be feeling quite so positive about BA or telephone support or telephone supervision or whatever*, *so 100*% *I think it*’*s great*” CM105
	Facilitating communication	“*Something that is quite helpful*… *if a client*’*s got an issue*, *especially something that is about medication I will say you know*, “*why don*’*t you speak to your GP about that*?” *and I will say* “ *I will be writing to your GP just to let him know that this is what we*’*ve discussed*”, *so the client would go*, *I would write a letter on the other side as well*, *and it*’*s quite nice because the client would then come back and go* “*Oh yeah*, *the GP got your letter*” *and when I speak to the GP they say* “*Oh yeah the client did come back to me after what you said*” *so I think*, *it really does work*” CM104
		“…*there*’*s that sort of linking where the GP was linked in*, *and I think that he was really pleased that erm*, *he was actually able to have a conversation with me about the medication*, *because he was actually feeling stuck and I think* [*names CM*] *was feeling a bit stuck*…” S102
		“*a lot of the time I*’*ve also noticed that through the GP if you do mention that through supervision what I have been told is X*, *Y and Z*, *then they could be*, *you know*, *they could be more likely to listen as well*, *to accept your opinion*, *so yeah*, *I think that works quite well as well*, *if you do tell them* ‘*after discussing this in supervision*, *this is what we thought*…” CM104
		“*I*’*ve had very little*, *if any involvement with the study except notification from you that a particular patient has been included on the study*” GP004
	Enhanced supervision	“*I think sometimes I*’*ll write to them asking them something or asking their opinion of something*, *then the GP will kind of contact me*, *get back to me*, *and I think on one or two occasions I have had a GP ringing just to ask if I*’*d seen a client or when am I next seeing a client*, *so yeah*, *I think that*’*s the only thing*, *it*’*d not something that happens that often*, *one or two occasions*” CM104
	Communication vs. collaboration	“*It*’*s such a big problem*, *I*’*m not blaming anybody because GPs don*’*t have the time*.... *You could try to make it happen*, *it would be nice just to see that*, *increasing that contact*… *it sounds like a very desirable thing that would be helpful for everybody*…*I think collaborative is too strong a word for collaborative care*, *it*’*s not truly collaborative in my opinion*, *but that*’*s my opinion*” S105
Catering for complexity	Recognition of complexity	“*I don*’*t think there is such a thing as pure depression*, *it comes in a package with lots of other things so when I say comorbid things*, *very often comorbid psychiatric problems*, *but also physical problems and never to forget*, *lots of social problems around*, *so you*’*ve got those three things there that are all competing*, *so there is a person with depression but at the same time there is obsessive compulsive disorder*, *or query*, *you know*…” S104
	The need to avoid mind-body dualism	“*I think that would be really helpful actually*, *for us to have more understanding of physical health problems and how they affect people*… *we need to recognize physical health problems and long term conditions and how they affect people*…*I think knowledge about those is really important*, *we just need to know more*” CM106
		“*I would have thought logically yes*, *it*’*s likely to be those sorts of people*, *the more complex your problem the more likely you are to benefit from it*, *erm*, *yeah*, *I would say comorbidity*, *absolutely*” GP015
	Usefulness of a collaborative care approach for people with complex problems	“*I think that the whole thing about collaborative care isn*’*t about the interventions*, *it*’*s actually about the system*, *and so that case management role is great*… *you know*, *I guess if you*’*re saying*, *well there*’*s the system which is about active follow up*, *is absolutely right and that covers all of these people*” S101

The main body of the results are presented using the NPT concepts of coherence, cognitive participation, collective action and reflexive monitoring with respect to the implementation of CC. Data is presented to support analysis and labelled by identifier and number (CM = case manager, S = supervisor; GP = general practitioner).

### Understanding the collaborative care framework (coherence)

Behavioural activation (BA), which formed the psychological intervention component of Colloborative Care in this study, was described by CMs as a user-friendly intervention and easy to understand, not just for themselves as practitioners, but also for the patients, as they didn’t find it “too over-complicated” (CM105). The CMs did find that the BA intervention encouraged them to develop joint plans with patients to a greater extent than in their usual practice:

“*By collaborative care what do I mean*? *Erm*, *I mean more that sense of working with the patient*… *and I think it*’*s more about reaching a shared understanding and working towards shared goals with enough input from other professionals that are involved in that person*’*s care*” *CM101.*

Supervisors and CMs understandably demonstrated a good understanding of the CC framework in addition to the intervention itself. For supervisors, this level of understanding was because of their role as co-investigators in the CADET trial. CMs reported that the CADET trial training had provided them with sufficient information and, opportunities to clarify and improve their understanding of CC, the intervention they were to deliver to patients, and the expectation of working with GPs. CMs described how their understanding of CC and their role had been changed by the training prior to working on the CADET trial:

“*I*’*d assumed* [*CC*] *would be self*-*help based stuff because we were primary care*, *and collaborating with other professionals. Since doing the training it*’*s mainly GPs that I*’*ve learned*, *but I kind of had the idea that it would be collaborating with other mental health workers*, *but not specifically GPs*” *CM103.*

Only a minority of GPs demonstrated a good understanding of CC, either due to their self-declared interest in mental health or previous experience of working within a CC framework.

“*So we*’*ve got more likelihood of being aware of what*’*s happening in terms of the management and then that can affect any input that we might have*, *say in terms of medication if we*’*re treating patients with antidepressants*, *we can get a feel for whether things were moving in the right direction and get the therapists*’ *input as well as our own assessment. So it can potentially improve our understanding of how the patient is progressing and responding and aid our management*” *GP010*.

However, the majority of GP respondents did not fully understand the CC framework and could not differentiate between the management of patients with depression in CC as distinct from routine care. As a result, some GPs used the qualitative interview as an opportunity for further clarification, perhaps suggesting a lack of such opportunities during their initial discussions about involvement in the trial.

“*GP014*: … *Are you able to define collaborative care for me so I know what you*’*re talking about*, *or not*?.

*Researcher*: *Erm*, *I mean what we*’*re trying to do it get an understanding of your understanding of it*, *so if you*’*re not aware*.

*GP014*: *I mean they*’*re all buzz words*, *so collaborative care*, *what it actually means*?”.

Some GPs described the main benefit of participating in the CADET trial as the potential for increased support in their management of patients with depression in the context of limited access to psychological therapy services to which to refer patients.

“*The CADET trial offered to me a resource which I thought would be beneficial. Another opportunity for somebody else to look at these patients*, *talk to them and share the workload in a way*, *with me*” *GP011*.

The GP is not reflecting specifically on the CC framework rather she seems to be reflecting on the benefits of participation in any trial where patients can access an additional ‘service’. Most GPs identified the potential benefits of adopting a more collaborative approach to patient care, particularly in patients with more complex problems.

“…*it*’*s likely to be those sorts of people*, *the more complex your problem the more likely you are to benefit from it*, *erm*, *yeah*, *I would say comorbidity*, *absolutely*… *the more complicated the things are*, *the more likely it is that the collaborative approach is going to help*” *GP015.*

It was not clear, even with probing in the interview, what GPs actually meant by a ‘collaborative approach’ and GPs were not clear whether a CC intervention would fit with their existing ways of working.

### Establishing relationships (cognitive participation)

A number of new relationships needed to be established in order to work within the CC intervention. Supervisors and CMs reported well-structured, weekly scheduled supervisory sessions which were arranged as part of the trial. Supervisors and CMs reported the value of an initial face-to face meeting to establish the relationships, followed by weekly telephone supervision. Supervision was also supported by the Patient Case Management Information System (PC-MIS), a web-based patient management system, demonstrating evidence of the work carried out for both establishing and sustaining collaboration between these two parties.

“*The supervision has been excellent I must say. It’s really nice to have it weekly, and it’s great to have PCMIS because it means we’re both looking at the same screen, so it’s been really good*” CM103.

Supervision as part of CADET was also considered by CMs as ongoing learning, affirming to their practice and confidence boosting:

“…*they might point stuff out to me or they might anticipate problems before they arrived which in my lesser experience maybe wouldn*’*t have foreseen so therefore they gave me some advice about how I might manage certain situations or what I might say to prepare a patient for something*, *erm*, *so yeah*, *it was fantastic*, *really*, *really good*” CM105.

Supervisors also acknowledged that supervision in CADET was superior to usual care, and highlighted the importance of such supervision to the success of CC, with one describing it as an “*integral part of*…*the whole collaborative care process*” (S102).

However, supervisors identified potential problems around identifying the right people to provide supervision outside of the research study, including finding people who are both willing and able to provide the same level of supervision as was delivered on the CADET study.

“*I think the biggest issue is the amount of supervisor time*, *and I think that*, *I think that we*’*re fairly generous in CADET in that the same supervisor is involved in following people up*, *and that means that you do get*, *that means that people do get really good supervision*, *but it*’*s quite*, *there*’*s quite a lot of time involved in that*… *It*’*s not that there*’*d be less time*, *there*’*d be less people that*, *erm*, *that are used to doing that kind of supervision*…” (S102).

In contrast, there was limited evidence of new relationships being established between the CMs and GPs in participating practices. Any liaison between CMs and GPs consisted of written information from the CM to practice, with direct contact unusual and only reported to have occurred when risk was deemed high, with few reports of CMs having direct access to the practice IT system:

“… *every four weeks we send a review letter*, *obviously you send the initial assessment letter to say* ‘*we*’*ve assessed this person*, *their main problem is*, *their scores are*’ *and then follow up letters every four weeks*” *CM105*.

“*Researcher*: *Have you been able to access to the patient records*, *has there been a sharing of information*?.

*CM103*:… *Erm*, *there*’*s a couple where I*’*ve needed to*, *and I can*’*t remember what practice it was but I went there and she said I had to send them a letter*, *so I had to come back here to fax them and then they faxed me a letter back*, *it was a bit*, *kind of ridiculous*”.

One CM did report having access to the patient records at some GP practices, but encountered different IT systems in different practices which was initially problematic, and she reported that developing good relationships with the practice administrative staff was essential to enable utilisation of these.

“*the other barrier I had was using the different computer systems in different surgeries*, *so that was dead complicated*, *but I got past that*, *and I found the staff were great because they*’*d just come and sign you on and things like that*, *because I couldn*’*t remember the password*” *CM102*.

As CMs were already working within existing services and were seconded to the CADET trial, a minority of CMs described pre-existing relationships with GPs which they found beneficial to engaging GPs in the CC framework. CMs also described a number of strategies they had attempted in order to enhance opportunities for collaboration with GPs, including identifying the GPs’ preferred method of communication at the beginning of the trial in anticipation of the need to communicate with GPs when working within a CC framework.

“*Initially with the study*, *what I did was*, *I went out and visited the GPs*…*and just said* ‘*what*’*s the best way for communicating*?’… *so it*’*s looking at what*’*s best for that GP*, *you know if you do get a relationship with them*” *CM102*.

Data suggests that the work carried out around setting up supervision and establishing the CM-supervisor relationships was important and appreciated by both parties. However, direct contact between CM and GP seemed to be the exception, rather than the rule, and at a time of crisis for an individual patient. Additional work was needed by the CM, and building on prior knowledge of the practice, to establish a working relationship with the GP, which would enable engagement as a routine.

### Working within a CC framework (collective action)

CMs identified few difficulties in delivering the psychosocial intervention with the patient, rather, they focused on the difficulties encountered in liaising or collaborating with GPs. Despite CMs reporting sending regular summary letters to GPs, the majority of GPs reported limited or no communication with CMs. It is unclear therefore if GPs did not receive these letters, or if they did not have time to read them.

“*I*’*ve had very little*, *if any involvement with the study except notification from you that a particular patient has been included on the study*” *GP004*.

“*I don*’*t think I had any contact personally with the case manager. I think I saw a letter or two*, *but no sort of telephone or email or anything of that sort*” *GP007*.

Either way, the limited communication reported by some GPs may account for their lack of awareness of the involvement of the CMs in the trial and the work that was being done with their patients.

“*Researcher*:…*You said that there would be someone with more specialist interest might be involved*, *erm*, *did you know who else was going to be involved*?.

*GP014*: *Recruiting patients*?.

*Researcher*: *Erm*, *so the person you would be collaborating with*? *GP014*: *No*.

*Researcher*: *No. OK. Erm*, *and so*, *are you aware now about the case managers that were involved in the study*? *That was involved in seeing the patient therapeutically*?

GP014: No.”.

The lack of GP involvement is supported by some CMs’ reports that although GPs were helpful once they had managed to contact them, GPs rarely initiated contact, which left CMs feeling that communication was one-sided.

“*since I*’*ve been working here*, *and that*’*s been two years now*, *I think I*’*ve only ever had GPs initiate contact with me twice. Yep. It*’*s really*, *really rare*, *which is a shame really*” *CM105*.

Despite the difficulties identified in contacting GPs, CMs reported improved relationships with participating GPs, along with identifying the benefits of this.

“*Yeah*, *I think*, *I mean there are some GPs who are really difficult to get hold of or*, *you do write to them and you don*’*t get a response and you have to try to chase them up*, *but a lot of the time what I have found is that they are quite helpful*, *you know*, *certain GPs are very easy to talk to on the phone*, *or make appointments with*, *so that*’*s been quite helpful*, *and erm*, *yeah*, *kind of discussing the patient as well*, *it*’*s*, *you know*, *I can suggest something*, *they can give me their side of what they*’*re doing*, *again*, *come to some sort of conclusion*…” *CM104*.

Some CMs suggested that co-location within GP practices could bring more opportunities for collaboration with GPs because of the increased possibility of informal communication, and compared this to their previous ways of working:

“*in the old days if we worked at a surgery*, *based there*, *it*’*s that relationship building that you have a chance to do*, *erm*, *and so at the moment we don*’*t do that as part of normal care*, *it*’*s harder to do*, *I think it*’*s impossible to do really*, *so what we get is*, *we*’*re based at one health centre and we get people from all different surgeries being referred through to that one health centre so we don*’*t get a chance to build those relationships*” *CM102*.

Supervisors recognised the difficulty achieving true collaboration between CMs and GPs:

“*I mean you*’*ve got to have people together to collaborate*, *you know*, *I just wonder to what extent this really is collaboration*, *because it*’*s only collaboration in name*, *in a way and the interested parties don*’*t really get down and talk to each other very much*… *It*’*s such a big problem*…*I*’*m not blaming anybody because GPs don*’*t have the time*.... *You could try to make it happen*, *it would be nice just to see that*, *increasing that contact*… *it sounds like a very desirable thing that would be helpful for everybody*…*I think collaborative is too strong a word for collaborative care*, *it*’*s not truly collaborative in my opinion*, *but that*’*s my opinion*” S105.

The supervisors recognised that the CC framework did not seem to fit within existing working practices of GPs.

Probably because of the set-up and frequency of supervision, supervisors and CMs reported good professional relationships with each other. Supervisors and CMs reported being impressed with each others’ skills, suggesting confidence in each others’ abilities. More specifically, supervisors reported satisfaction with the CMs’ skills for delivering BA within a CC framework, even to those patients identified as complex.

“*I*’*ve been pretty impressed by the ability of the case managers to assess and manage some people who have not always been that straight forward*, *by any means*, *and these are people who are supposed to have*, *you know*, *these are people who have I suppose moderate degrees of depression*, *but they*’*ve got complicated life problems as well*, *some of them have been in crisis*, *and they*’*ve managed them. I think it*’*s gone pretty well*” *S102*.

Likewise, CMs were enthusiastic by what they considered to be enhanced supervision, because of its increased frequency and the supervisors’ wealth of experience and knowledge.

“*they might point stuff out to me or they might anticipate problems before they arrived which in my lesser experience maybe wouldn*’*t have foreseen so therefore they gave me some advice about how I might manage certain situations or what I might say to prepare a patient for something*, *erm*, *so yeah*, *it was fantastic*, *really*, *really good*” *CM105*.

There was little evidence in the GP data that the work conducted by the CMs and supervisors had any impact on the GPs’ routine consultations, or their work with patients.

“…*as far as the CADET study is concerned*, *we*’*ve not*….*it*’*s happened alongside us really*, *it hasn*’*t had*…*it certainly hasn*’*t been detrimental to anything that we*’*ve been doing*, *but that*’*s not really what I mean. What I mean is that we identified patients but then didn*’*t need to change what we were doing very much*” *GP007*.

CMs reported that they had taken or planned to take many elements from CC (such as increased collaboration with GPs and medication management, as well as the BA psychosocial intervention) back into their routine work, which demonstrates that this approach is acceptable to CMs and has the potential to become normalised within their routine practice.

“*What I will probably take back is a lot more information on medication*…*when I was working prior to that* [*CADET*], *the focus wasn*’*t so much on the medication*, *yeah*, *and I don*’*t think that I had much idea of medication*, *and I think now*, *there was a time when I wasn*’*t too keen on medication myself*, *I wasn*’*t too sure if medication really worked*, *whereas now I*’*ve seen that it is quite helpful so I would probably emphasise the medication with my patients*, *yeah*, *and I probably will take the whole BA in terms of being active and how that helps with the depression*, *so yeah*, *as a whole*, *the whole thing*, *but if there*’*s one thing I*’*m going to focus on more it*’*ll be the medication*, *yeah*” *CM104*.

Our data suggest that changes in organisation within practices would be required to establish relationships between CMs and GPs and facilitate successful collaboration, such as integrated IT systems and enhanced opportunities for GP/CM communication and possibly co-location of professionals. CC would need to be seen as fitting in with the routine work of the practice in order for GPs to make changes to accommodate the work involved.

### Evaluating collaborative care (reflexive monitoring)

The weekly supervision presented regular opportunities for CMs and supervisors to reflect on patients and monitor their progress jointly. CC, and the psychosocial intervention, was described as effective and acceptable by CMs and supervisors; although it seems that CMs reflected on the perceived effectiveness of the psychosocial intervention (which formed the majority of their work with individual patients), rather than the CC framework as a whole. The CMs described how they monitored patients through both the collection of routine data (the Hospital Anxiety and Depression Scale [HADS]), their own perception of the patients’ progress and discussions within supervision:

“…*a couple of people who*, *especially one*, *he*’*s had long standing social anxiety so a bit more of a complicated problem*, *but also depression*, *and we just worked away on the depression and we saw an improvement*, *so just by doing that behavioural activation*, *so sometimes even though someone*’*s got more complex problems*, *for some people behavioural activation just saw quite an improvement*, *you know*” *CM102*.

“*I think it*’*s effective*… *I think that has been the most satisfying part*, *that I know it can work*, *I*’*ve seen BA work*” *CM105*.

Although CMs and supervisors identified some problems around delivering the trial psychological intervention (BA) in line with the protocol for those with comorbid mental health and complex social problems, the principles of intervention were still perceived to be acceptable in reducing symptoms of depression.

“*I think I would*’*ve liked to work on anxiety a bit more*, *but at the same time*…*we*’*ve watched those depression scores come down*” *CM101*.

Some GPs did report receiving positive feedback from the patients about their experience with the CMs and of the intervention (BA, MM), which led the GPs to believe there was some value in the intervention. This ‘second-hand’ knowledge was the only evidence on which GPs could reflect on the intervention, or on the CC framework.

“*A significant amount of them have reported personally that they have felt better after participating in the trial*, *in the study and then whatever the numbers there is some benefit in it*” *GP002*.

“*I think certainly with a number of patients they did seem to gain considerable benefit and their depression was improved and their general social functioning seemed to be improved*…*I didn*'*t get any negative feedback about the process*” *GP006*.

In contrast to the CMs’ reports, GPs reported that they did not actively seek feedback from patients regarding their experience of CC, and feedback was only received when volunteered by the patients.

“*Generally from the patients we have had very positive feedback*, *and often our patients are generally kind of if there is something they don*'*t like they will come and tell us*” *GP009*.

Similarly, some GPs suggested that the results of the trial rather than their views would determine their opinions on the future possibility of working in a new way:

“*one of my managers doesn*’*t see how*, *if CADET really works*, *so*, *and at the same time I*’*m not sure because I*’*m waiting*, *I look for the actual*, *you know sometimes I think it hasn*’*t worked*, *sometimes I think it has worked*… *I suppose that*’*s where the results will show*, *whether that*’*s worked*” *CM101*.

“… *we*’*re talking small numbers and I think we need to see some outcome data rather than just my anecdotal subjective views of possibilities*.” *GP010*.

The supervisors raised concerns about who would take on the responsibility of supervision of the CMs if CC was implemented into routine practice, because of both the expertise and time required to deliver supervision to the same standard and frequency as was delivered in the trial. CMs also identified time as the biggest resource necessary to implement CC, because of the time needed to maintain the prompt commencement of the intervention following referral, for the time required for the administration involved in communicating with GPs, and the time invested in supervision.

“…*I think the collaborative care part of it*, *because*, *writing a letter after assessment and then keeping a GP updated with letters*, *often what happens at* [*names team*], *the GPs are sent a letter on discharge with a summary of what happened*, *so that*’*s kind of like no collaboration at all*, *for a lot of people there*’*s absolutely no collaboration*, *and that*’*s just down to time really and just the number of patients that everybody has*” *CM106*.

GPs however felt that the main obstacle to implementing CC would be the financial cost of commissioning CC services, which they perceived would be more expensive than current care:

“*Researcher*: … *What are your views on whether collaborative care should be commissioned as a service for management of people with depression in primary care*?.

*GP005*: *I would say it is an excellent way forward. However*, *it couldn*’*t really have come at a worse time could it*?.

*Researcher*: *Could you explain that*?

*GP005*: *Well in terms of all the financial restrictions and all the changes that are going to be happening at the moment*.

Thus, CMs and Supervisors valued the case manager role with expert supervision as well as the specific psychological intervention including behavioural activation and medication management components. CMs placed less emphasis on the liaison between CM and GP. GPs did not report actively reflecting on and monitoring the collaborative aspect of CC, between CMs and GPs, but CMs described examples of liaison and how it might be facilitated. CMs were positive about implementing CC into routine practice. Although possibly the emphasis was on the psychosocial intervention, rather than the broader CC framework; but lack of time, concerns over supervisory arrangements in routine practice and perceived cost of implementation were identified by all participants as barriers to this.

## Discussion

### Summary of main findings

We found that although case managers and supervisors regarded collaborative care as a coherent practice, the collective action required to implement collaborative care was made difficult by GPs’ lack of understanding of the CC framework.

Although professionals reflected positively on the potential or experienced benefits of implementing a CC approach (reflexive monitoring), the apparent lack of GPs’ understanding meant that they were more passive in their roles than CMs and supervisors, and therefore reported that the CC model as implemented had impacted little on their routine work or professional relationships. The lack of reported contact between GPs and CMs was identified as a frustration for CMs, and resulted in little opportunity for GPs to increase their knowledge of CC pragmatically, which may account for the reported limited impact on their usual practice. This suggests that more work is required to ensure GPs fully understand their roles within the CC framework in order to successfully embed CC within GPs’ routine practices, either taking a didactic approach by providing training and/or a protocol for GPs, as was provided for CMs and supervisors in the CADET trial, or through facilitating the CM-GP collaboration further to provide more opportunities for pragmatic learning. Results suggest that such communication may be facilitated by setting up multi-site access to information systems or patient records in the absence of co-location of professionals. We also suggest that building in opportunities for formal communication between CMs and GPs into the CC framework would facilitate professional collaboration, and may in turn increase GPs’ understanding of the CC framework and their roles within it.

### Strengths and limitations of the study

The strengths of this study lie in the multi-professional viewpoints obtained, adding to previous work [22] by exploring GP perspectives on the intervention. As this previous qualitative work around implementing CC into UK primary care used the Normalisation Process Model (NPM), this analysis builds on this work by including analysis around the additional three constructs of NPT (Coherence, Cognitive Participation and Reflexive Monitoring) which are not included in NPM. Data was discussed and analysed by a multi-professional team (primary care, nursing, psychology) which contributes to trustworthiness of analysis.

There are some limitations to this study. Although purposive sampling of GPs was attempted, GPs were difficult to recruit to this qualitative study, with a majority of those who refused citing lack of time or limited involvement in the trial as reasons for this. However, category saturation was achieved within the data, although the difficulties of recruiting GPs may mean that the data may not represent the views and experiences of GPs in all participating practices.

GP interviews were completed by telephone, while CM and supervisor interviews were face-to-face, which may account for GP interviews being shorter in length and possibly less detailed. It is possible that the length of GP interviews was influenced by the interviewers being non-clinicians [29], but GPs interviewing fellow clinicians can have a negative effect on responses through causing respondents to feel as though their professional knowledge and practice is under scrutiny [30,31]. The supervisors interviewed in this study were also co-investigators on the CADET main trial, therefore their views are likely to framed by their academic investment in the study. As CADET trial researchers conducted the interviews, some researcher bias may be evident as this is likely to have affected the participants’ responses, particularly the supervisors and CMs [32]. We attempted to interview across sites, to reduce this bias. At times during interviews with CMs, respondents seemed to find it difficult to reflect on the CC framework, referring instead to the psychological intervention they were delivering to patients; their positivity about working with patients was greater than their experiences of relating to GPs.

### Comparison with existing literature

This study was nested in the CADET trial which showed that CC is an effective intervention for depression [19] and supports previous studies which highlighted the need for clear arrangements for the liaison between GPs and CMs [21]. More specifically, our study supports previous findings that in order to facilitate professional liaison, there may be an advantage to the mental health practitioners involved in delivering CC being co-located within GP practices, and priority should be given to improving sharing of information between the professionals involved [29,30]. The value of enhanced supervision within a CC framework identified in this study was also emphasised in a USA study of case management [33]. The effect size in the CADET trial [19] was similar to trials of other, psychological interventions; we might hypothesise that enhanced GP-CM liaison might have increased the effectiveness of the CC intervention.

### Implications for future research and clinical practice

The value of utilising the NPT framework to analyse implementation from multiple perspectives is illustrated by our approach [34]. The limited liaison between GPs and CMs reported suggests that more work is needed to facilitate collaboration around individual patients. Some structural aspects were identified which may facilitate liaison including; shared place of working, shared IT systems, facilitating opportunities for informal meetings and building in formal collaboration into the CC framework. However, although considered by many to be desirable, it is unclear if such an increase in collaboration between CMs and GPs would improve clinical outcomes. The extent to which additional collaboration between CMs, supervisors and GPs beyond established communication lines, despite being a core component of the CC model, is actually necessary for effective patient management of depression is as yet undetermined. Whether a CC framework can be adopted in a more cost effective and sustainable way, without the emphasis on CM-GP liaison, should be a future research question.

## Conclusion

Collaboration around the management of patients with depression in primary care was valued by professionals, particularly for managing patients with more complex problems, but GPs’ understanding of CC and long-standing organisational barriers hindered communication. In order to successfully embed GP participation into collaborative care models in primary care, further work is needed to address these organisational barriers to facilitate professional collaboration such as improving sharing of information in the absence of co-location. An increase in communication may also provide a pragmatic approach to increasing GPs’ understanding of the CC framework and their roles within it. Didactic and pragmatic approaches are not mutually exclusive, and a combination of these approaches may be the best way to improve GPs’ understanding of, and involvement in a collaborative approach to depression care. The clinical impact of any enhanced communication would have to be tested further. Enhanced supervision, as reported in this study may be collaboration enough to result in improved patient care. It is important to find the most cost-effective approach to the management of patients with depression in primary care, given the cost implications or new models of care, and the current financial climate.

## Abbreviations

BA: Behavioural activation; CC: Collaborative care; CM: Case manager; GP: General practitioner; IAPT: Increasing access to psychological therapies; MM: Medication management; NICE: National Institute for Health and Clinical Excellence; NPT: Normalisation process theory; NPM: Normalisation process model; RCT: Randomised controlled trial.

## Competing interests

The authors declare that they have no competing interests.

## Authors’ contributions

DR, CCG, LG, NC, PS and EA were involved in the design of the study. NC, EA and PS were responsible for data collection, and led by CCG, conducted the data analysis. All authors were involved in drafting the manuscript and provided critical feedback on earlier versions of the paper. All authors read and approved the final manuscript.

## Pre-publication history

The pre-publication history for this paper can be accessed here:

http://www.biomedcentral.com/1471-2296/15/78/prepub
